# Complete genome sequence of multidrug-resistant *Raoultella terrigena* strain RT01-5M1 isolated from farmed salmon in Canada

**DOI:** 10.1128/mra.00153-24

**Published:** 2024-06-27

**Authors:** Bei Zhang, Tianbi Tan, Derek D. N. Smith, Marc-Olivier Duceppe, Bashudev Rudra, Radhey S. Gupta, Dele Ogunremi

**Affiliations:** 1 Ottawa Laboratory Fallowfield, Canadian Food Inspection Agency, Ottawa, Canada; 2 Department of Chemistry, Carleton University, Ottawa, Canada; 3 Ecotoxicology and Wildlife Health Division, Environment and Climate Change Canada, Ottawa, Canada; 4 Department of Biochemistry, McMaster University, Hamilton, Ontario, Canada; University of Southern California, Los Angeles, California, USA

**Keywords:** *Raoultella terrigena*, antimicrobial, multidrug, salmon, aquaculture

## Abstract

The genome sequence of multidrug-resistant *Raoultella terrigena* RT01-5M1 strain isolated from Canadian farmed salmon was determined using Oxford nanopore and Illumina MiSeq sequencers. The assembled chromosome was estimated at 5,699,993 bp in size, with two plasmids, 164,879 bp and 82,046 bp. The chromosome and smaller plasmid contained antimicrobial resistance genes.

## ANNOUNCEMENT

*Raoultella terrigena* causes primary and opportunistic hospital-acquired diseases (1, 2). Here, we present the genome of *R. terrigena* RT01-5M1 strain isolate from a surface mucous swab from an aquacultured Atlantic salmon obtained in 2022 from British Columbia, as part of a Canadian government regulatory program exempted from animal experimentation ethics review. Mucus suspension (100 µL) grown in Brain Heart Infusion (BHI) broth (1 mL) for 2 days at room temperature was plated (200 µL) on a blood agar plate (BAP) containing 100 µg/mL ampicillin. A pure colony (RT01-5M1) obtained after re-streaking on BAP containing 100 µg/mL tetracycline and purified using antibiotic-free BAP was cultured in BHI media and glycerol stored at −80°C. Separate inocula of RT01-5M1 were grown in BHI media for DNA extraction (Wizard Genomic DNA purification kit; Promega, Madison, WI, USA) and quantification (Spectrophotometer DU 730, Beckman Coulter, Mississauga, ON, Canada, and Qubit Flex fluorometer, ThermoFisher Scientific Inc., Carlsbad, CA, USA) without shearing or size selection for library preparation.

Two short read libraries (DNA Tagmentation, Illumina, San Diego, CA) sequenced on the Illumina MiSeq were deposited in the GenBank as a single accession (SRR27955936) and merged with a third run (SRR27918695) from a separate DNA library to obtain 2 × 4,165,016 reads and a coverage of 208X. Two long read libraries (native DNA, SQK-LSK109 ligation kit) were barcoded and sequenced with R9.4.1 flow cells (MK1C device; Oxford Nanopore Technologies Inc., Oxford, UK) and merged (65,412 reads; 110X coverage, NG50 = 10.6 Kb). Multiple sequencing rounds ensured a high-quality genome assembly.

Basecalled nanopore reads (Guppy v6.5.7, super accuracy mode), merged, and adapter-trimmed with Porechop v0.2.4 (https://github.com/rrwick/Porechop), were filtered to retain long reads (Filtlong v0.2.1; https://github.com/rrwick/Filtlong) and assembled with Trycycler v0.5.4 (3) to generate a consensus long read with the aid of Raven v1.8.1 (4), Shasta v0.8.0 (5) and Flye v2.9 (6). Trimmed short reads (fastp v0.23.4; 7) were used to polish the long read assembly (NextPolish v1.4.1, ntEdit v1.3.5 and Polypolish v.0.5.0) (8–10). The circular chromosome and plasmids generated were annotated with PGAP v2023-10-03.build7061 (11). The plasmids were confirmed by homology search using blast on the nr database (accessed on 2024–03-27). Long and short read coverage was established using minimap2 v2.24 (12), SAMtools v1.15.1 (13), and qualimap v2.2.2 (14). Default parameters were used for all software unless otherwise specified.

*R. terrigena* RT01-5M1 genome consisted of a 5,699,993 bp chromosome (57.5% GC) and two plasmids, 164,879 bp (52.4% %GC) and 82,046 bp (53.7% GC) and 5,732 genes. The genome was 99.99% complete (CheckM; 15). Species identification was achieved using standard threshold procedures for species boundary (16), including 16S rRNA similarity (99.87%; https://www.ezbiocloud.net/tools/contest16s, 17), average nucleotide identity (97.42%; https://www.ezbiocloud.net/tools/ani, 18); mashID combined with proGenomes v3 (98.7%; https://github.com/duceppemo/mashID, 19) and genome-to-genome distance (77.6%) compared with *R. terrigena* type strain NBRC 14941. Antimicrobial resistance gene was detected with ResFinder v4.1.5 (20), and nine were found on the smaller plasmid and two on the chromosome (Table 1). RT01-5M1 demonstrated multidrug resistance against a panel of antibiotics using the Sensititre procedure (ThermoFisher Scientific).

**TABLE 1:** Antimicrobial resistance profile of *Raoultella terrigena* RT01-5M1^*a*^

Antibiotic resistance	Phenotype	Genomic location of AMR genes		
		AMR gene	Chromosome 5.7 MB	Plasmid 82 Kb
Chloramphenicol	R	*floR*	—^*b*^	35458….39671
Penicillin	R	*bla*_TER-2_	2670817….2671671	—
Streptomycin	R	*aph(6)-Id* *aph(3”)-Ib* *aadA2*	— — —	21554….22390 36665….37501 22390….23192 35862….36665 28487….29278
Sulphonamide	R	*sul1* *sul2*	— —	29783….30622 34986….35801
Tetracycline	R	*tet(D)*	—	45552….46736
Trimethoprim	R	*dfrA12*	—	27582….28079
Chlorhexidine & cetyl- primidine	R	*qacE*	— —	— 29442….29723
Fosfomycin	NT	*fosA*	5029949….5030368	—
Erythromycin	R	ND	ND	ND
Daptomcyin	R	ND	ND	ND
Linezolid	R	ND	ND	ND
Quinupristin/dalfopristin	R	ND	ND	ND
Vancomycin	R	ND	ND	ND

^*a*^ NT, not tested; ND, not detected.

^*b*^ "—" denotes the absence of the gene in the respective molecule, either the chromosome or the plasmid.

## Data Availability

All sequence data were submitted to GenBank under BioProject ID PRJNA1073544. Raw reads from Nanopore were deposited in the Sequence Read Archive under accession numbers SRR27946164 and SRR27918694 while Illumina raw reads had SRR27955936 and SRR27918695. The chromosome, small plasmid, and big plasmid have the following respective accession numbers: CP145801, CP145802, and CP145803.
